# The influence of patient perception of physician empathy on patient satisfaction among attending physicians working with residents in an emergent care setting

**DOI:** 10.1002/hsr2.337

**Published:** 2021-08-17

**Authors:** Ryan Kirby, Heidi C. Knowles, Anant Patel, Naomi Alanis, Colton Rice, James P. d'Etienne, Chet D. Schrader, Nestor R. Zenarosa, Hao Wang

**Affiliations:** ^1^ Department of Emergency Medicine John Peter Smith Health Network Fort Worth Texas USA

**Keywords:** attending physicians, empathy, residents, satisfaction

## Abstract

**Background:**

It is unclear whether the patient's perception of attending physician empathy and the patient's satisfaction can be affected when attending physicians work alongside residents. We aim to determine the influence residents may have on (1) patient perception of attending physician empathy and (2) patient satisfaction as it relates to their respective attending physicians.

**Methods:**

This is a prospective single‐center observational study. Patient perception of physician empathy was measured using Jefferson Scale of Patient Perception of Physician Empathy (JSPPPE) in both attendings and residents in the Emergency Department. Patient satisfaction with attending physicians and residents was measured by real‐time patient satisfaction survey. Multivariate logistic regressions were performed to determine the association between patient satisfaction and JSPPPE after patient demographics, attending physician different experience, and residents with different years of training were adjusted.

**Results:**

A total of 351 patients were enrolled. Mean JSPPPE scores were 30.1 among attending working alone, 30.1 in attending working with PGY‐1 EM residents, 29.6 in attending working with PGY‐2, and 27.8 in attending working with PGY‐3 (*p* < 0.05). Strong correlation occurred between attending JSPPPE score and patient satisfaction to attending physicians (*ρ* > 0.5). The adjusted odds ratio was 1.32 (95% CI 1.23‐1.41, *p* < 0.001) on attending's JSPPPE score predicting patient satisfaction to the attending physicians. However, there were no significant differences on patient satisfaction among four different groups.

**Conclusion:**

Empathy has strong correlation with patient satisfaction. Decreased patient perception of attending physician empathy was found when working with senior residents in comparison to working alone or with junior residents.

## 1 INTRODUCTION

Empathy is the ability to understand or feel what other persons' are experiencing and becomes an important indicator of building up provider‐patient rapport in clinical practice.^1^, ^2^ Better patient‐centered care with improved clinical outcomes have been associated with positive perception of empathy among health care providers.^3^, ^4^ Empathy can be measured either by health care providers themselves or by their patients.^5^, ^6^ High consistency has been reported between patient perception of provider empathy and patient satisfaction to the providers.^7^, ^8^ By far, one of the commonly used empathy measures is the Jefferson Scale of Patient Perception of Physician Empathy (JSPPPE).^5^, ^9^

JSPPPE is a well validated tool to measure health care providers' empathy consistent findings.^10^, ^11^, ^12^ However, the use of JSPPPE in the field of Emergency Medicine (EM) has been less studied. Previous studies have considered that inconsistent findings in measuring empathy among health care providers working in the Emergency Department (ED) might be due to providers having less time to spend with the patients, resulting in suboptimal patient‐provider rapport.^13^ In addition, ED providers rarely follow up with their patients and this lack of patient engagement could further prevent them from building up routine patient‐provider rapport.^14^

On the other hand, the common practice model of an academic teaching hospital ED will have attending physicians working with residents of different training levels. Attending physicians have responsibilities of taking care of their patients while educating residents. By far, we are uncertain of whether working with residents will affect patient perception of attending physicians' empathy, thus subsequently affecting patient satisfactions to the attending physicians. It is also largely unknown whether residents' empathy and patient satisfaction toward residents can directly affect patient satisfaction toward attendings.

Determining the association and interaction of patient perception of provider empathy and patient satisfaction among attending physicians and residents is very important because it provides evidence of improving patient centered care while also helping improve resident education. A better understanding of the attending‐resident practice pattern and how it affects patient centered care can provide guidance to contribute building an optimal clinical practice curriculum among different specialties with residency programs.

In this study, we aim to (1) investigate the association between patient perception of provider empathy when the EM attending physicians worked with residents of different training years; and (2) to determine whether attending physician empathy and satisfaction is affected by working with residents.

## 2 MATERIAL AND METHODS

### 2.1 Study design and setting

This is a secondary data analysis derived from a single‐center prospective observational study in an urban hospital ED.^13^ The study hospital is a Level‐1 trauma center, a comprehensive stroke center, chest pain center, and tertiary referral center. The study ED sponsors an ACGME (Accreditation Council for Graduate Medical Education) accredited 3‐year EM residency program and has an annual patient volume of more than 125 000. This study was approved by the local Institutional Review Board (IRB#1352504‐6) and was performed under the Helsinki research ethics statement. All the participants signed the informed consent form.

### 2.2 Study participants

ED attending physicians, EM residents, and ED patients who consented and agreed to participate in this study were included. From January 2019 to August 2019, patient perception of physician empathy and patient satisfaction surveys were given to all patient participants who agreed to participate, using either paper‐ or tablet‐based platform, before they were discharged from ED. We excluded subjects who: (1) declined to participate; (2) completed less than 20% of study survey questions; (3) were evaluated by a physician (either attending physician or resident) who did not participate in this study; and (4) were unable to identify their attending physician/residents in order to complete the surveys.

### 2.3 Patient perception of physician empathy and physician satisfaction measurements

Jefferson Scale of Patient Perception of Physician Empathy (JSPPPE) was used for patient perception of physician empathy. JSPPPE can measure different health care providers' empathy including physician, nurses, residents, and medical students.^12^, ^15^, ^16^ It can be applied to physicians of different specialties including orthopedics, family medicine, and internal medicine.^15^, ^17^, ^18^ JSPPPE includes five questions, each of which were assessed using a 7‐point Likert Scale (“strongly disagree” = 1 to “strongly agree” = 7), with a total score ranging from 5 to 35. Higher JSPPPE scores indicate higher patient perception of physician's empathy. In this study, participating patients completed the JSPPPE on both the attending physicians and the residents separately if an individual patient was cared by both. If patients were cared by multiple attending physicians and residents, patients were offered to complete as many surveys as possible if they were able to differentiate individual providers' names or characteristics. Immediately upon completion of JSPPPE, a patient satisfaction survey was rendered. Patients were asked to score their satisfaction on the surveyed providers. Patient satisfaction was assessed using a 5‐point Likert Scale (“very dissatisfied” =1, “dissatisfied” =2, “neither satisfied nor dissatisfied” =3, “satisfied” =4, and “very satisfied” =5). Similarly, patient satisfaction was surveyed on both the attending physician and resident separately if an individual patient was cared for by both the attending and the resident.

### 2.4 Study protocol and study variables

For patient selection, a set of 4‐hour blocks were randomly generated every 3 months by using the random number generator in STATA (College Station, TX) as previously reported.^13^ JSPPPE and patient satisfaction surveys were only collected within randomly selected time‐blocks to avoid patient selection bias and Hawthorne effect. Study included demographics including attending physician, resident, patient age (three categories: <40); 40‐50; ≥50), races (Caucasian, African American, and others), gender (male vs female), and ethnicity (Hispanic vs Non‐Hispanic). Other variables included attending physicians' experience based on their number of years of practice after residency graduation (<5 years; 5‐10; 10‐20;  ≥20 years), resident different post‐graduate years (PGY) of training (PGY‐1, PGY‐2, and PGY‐3). Patients were also divided into four groups based upon patients who were cared for by (1) the attending physicians only; (2) both the attending physicians and PGY‐1 EM residents; (3) both the attending physicians and PGY‐2 EM residents; and (4) both the attending physicians and PGY‐3 EM residents. JSPPPE and patient satisfaction to both the attending physicians and residents were calculated and analyzed separately. Due to the skew data (>90% of patients rates satisfaction survey as either “5: very satisfied” or “4: satisfied), we further classified patient satisfaction into either “very satisfied” (satisfaction score of 5) or “not‐very satisfied” (satisfaction score of 0‐4) groups.

### 2.5 Data analysis

Data were initially analyzed including general demographics, JSPPPE scores, and patient satisfaction scores. Correlations among JSPPPE and patient satisfaction were measured using Spearman's Rho (*ρ*) test with (1) |*ρ*| ≥ 0.5 indicating strong correlation, (2) 0.5 > |*ρ*| ≥0.3 indicating moderate correlation, and (3) 0.3 > |*ρ*| ≥0.1 indicating a weak correlation. Multivariate logistic regressions were performed to determine the association between patients' satisfaction and JSPPPE to both the attending physicians and the residents separately with the adjustment of patient demographics, attending physicians' experience, and resident different years of training. A power analysis of sample size enrollment was previously reported.^13^ All analyses were performed using Stata v14.2 (College Station, TX).

## 3 RESULTS

**FIGURE 1:**
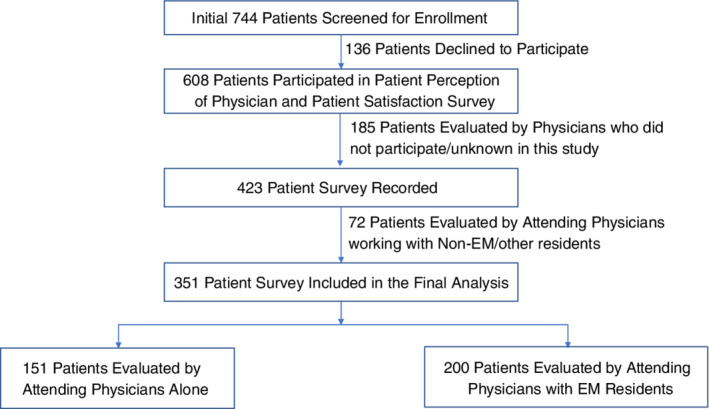
The study flow diagram

From January 2019 to August 2019, a total of 351 patients were enrolled in the final analysis. A detailed study flow diagram is shown in Figure 1. During the study period, 28 ED attending physicians and 33 EM residents participated in this study. Among 28 ED attending physicians, over 50% were male, non‐Hispanic White, and younger than 40, similar demographics as to EM residents (Table 1). Among 351 ED patients, 49% were male, over 50% were older than 50, and more Caucasian patients (44%) than African American patients (32%, Table 1). The mean scores of patient perception of attending physicians' empathy (JSPPPE, 29.5) and patient satisfaction score to attending physicians (4.5) were lower than those of the residents separately (31.0 and 4.6, *p* > 0.05).

**TABLE 1:** General information of study population

	Attending physician (n = 28)	EM resident (n = 33)	Patient (n = 351)
Demographics			
Age group – (n, %)			
<40 years	16 (57)	32(97)	102 (29)
40–50 years	7 (25)	1(3)	64 (18)
>50 years	5 (18)	0(0)	185 (53)
Sex (n, %)			
Male	19 (68)	24(73)	173 (49)
Female	9 (32)	9(27)	178 (51)
Race (n, %)			
Caucasian	22 (79)	26(79)	154 (44)
African American	1 (4)	0(0)	113 (32)
Asian	5 (18)	7(21)	1 (0.3)
Others			83 (24)
Ethnicity (n, %)			
Hispanic	2 (7)	0(0)	96 (27)
Non‐Hispanic	26 (93)	33(100)	255 (73)
Study measurements			
JSPPPE – Mean (SD)	29.5 (5.9)	31.0 (4.5)	
Patient satisfied with Physician – Mean (SD)	4.5 (0.8)	4.6 (0.7)	

Abbreviation: JSPPPE, Jefferson Scale of Patient Perception of Physician Empathy.

Strong correlation occurred between patient perception of attending physician empathy and patient satisfaction to the attending physicians (*ρ* = 0.5589, Table 2), while moderate correlation occurred among EM residents (*ρ* = 0.4677). In addition, strong correlation of patient perceptions of empathy occurred between the attending physicians and residents who worked together (*ρ* = 0.6003) indicating patient perception of empathy can be influenced by providers caring for the same patient. This pattern was not observed when investigating the correlation between patient perception of empathy and patient satisfaction (ie, weak correlation between patient satisfaction to attending and patient perception of resident empathy (ρ = 0.2926) and vice versa, *ρ* = 0.2982, Table 2).

**TABLE 2:** Correlations between patient perception of provider empathy and patient satisfaction in attending physicians and residents

	Attending JSPPPE	Attending satisfaction	Resident JSPPPE	Resident satisfaction
Attending JSPPPE	X			
Attending satisfaction	0.5589	X		
Resident JSPPPE	0.6003	0.2926	X	
Resident satisfaction	0.2982	0.4696	0.4677	X

Abbreviation: JSPPPE, Jefferson Scale of Patient Perception of Physician Empathy.

To determine the potential influence of resident training affecting attending physicians' empathy to patients and patient satisfaction, this study further divided patients into four groups. Mean JSPPPE score of patients who were evaluated by attending physicians alone (30.1) was similar in comparison to the mean JSPPPE score of patients who were cared for by attending physicians working with the junior EM residents (PGY‐1=30.1, Table 3). However, mean JSPPPE scores continuously decreased among patients who were cared for by attending physicians working with senior EM residents (attending with PGY‐2=29.6 and attending with PGY‐3=27.8, *p* < 0.05). As for patient satisfaction, strong correlation (*ρ* > 0.5) occurred when compared with the JSPPPE scores among all four groups. In addition, mean JSPPPE scores for residents were higher as compared to the mean JSPPPE scores for the attending physicians regardless of different resident training years. Moderate correlation occurred between JSPPPE and patient satisfaction to residents. Consistent lower JSPPPE and patient satisfaction scores (differences of the scores and its correlation) were found between attending physicians and EM residents who worked together (Table 3).

**TABLE 3:** Comparisons of patient perception of physician empathy and their satisfaction with physicians among attending physicians working with emergency medicine residents of different training levels

	Attending only	Attending with PGY‐1 EM residents	Attending with PGY‐2 EM residents	Attending with PGY‐3 EM residents	*P* value
Attending JSPPPE*	30.1 (5.1)	30.1 (5.5)	29.6 (5.9)	27.8 (7.1)	0.0316
Attending satisfaction	4.5 (0.8)	4.6 (0.7)	4.4 (0.8)	4.4 (0.8)	0.7173
Attending correlation	0.5182	0.6033	0.8300	0.7457	
Resident JSPPPE*		31.3 (4.4)	32.1 (3.7)	30.2 (4.9)	0.0416
Resident satisfaction		4.7 (0.5)	4.5 (0.8)	4.6 (0.6)	0.3826
Resident correlation		0.6339	0.4893	0.5886	
JSPPPE differences		−1.2 (4.7)	−2.3 (4.7)	−2.0 (6.1)	0.5628
Satisfaction differences		−0.1 (0.7)	−0.1 (0.7)	−0.2 (0.9)	0.7278
Difference correlation		0.6465	0.5620	0.7424	

Abbreviation: JSPPPE, Jefferson Scale of Patient Perception of Physician Empathy.

* *P* < 0.05.

A multivariate logistic regression analysis was then performed to determine the risks associated between patient satisfaction to attending physicians and patient perception of attending physicians' empathy after adjustment of the potential confounders (eg, working with residents, years of physicians' practice, patient demographics, Table 4). It showed that when patients' perception of attending physician's empathy was high, it increased the odds of higher patient satisfaction. While, working with/without residents, attending physicians' experience and patient demographics had no significant association with patient satisfaction (Table 4). In addition, the association between patient satisfaction to EM residents and their perception of residents' empathy was also analyzed using similar regression model. When patients' perception of residents' empathy is high, it increased the odds of higher patient satisfaction to residents, indicating a consistent correlation between that JSPPPE and patient satisfactions (Table 5).

**TABLE 4:** Risks associated with patient satisfaction among attending physicians

	Adjusted odds ratio (95% CI)	*P* value
Attending physicians		
Working alone	Reference	
working with residents	1.48 (0.83, 2.65)	0.182
Patient perception of attending physicians' empathy	1.32 (1.23, 1.41)	<0.001
Attending experience		
Less than 5 years after graduated from residency	Reference	
5–10 years (including 5 years) from residency	0.62 (0.23, 1.71)	0.356
10–20 years (including 10 years) from residency	0.31 (0.11, 0.89)	0.029
≥20 years after graduated from residency	0.86 (0.25, 2.98)	0.808
Patient age	1.01 (0.99, 1.03)	0.168
Patient gender		
Male	Reference	
Female	0.95 (0.54, 1.67)	0.860
Patient races		
White	Reference	
Black	0.45 (0.23, 0.88)	0.019
Others	1.08 (0.35, 3.31)	0.896
Patient ethnicity		
Not Hispanic/Latino	Reference	
Hispanic/Latino	0.45 (0.15, 1.35)	0.155

**TABLE 5:** Risks associated with patient satisfaction among emergency medicine residents

	Adjusted odds ratio (95% CI)	*P* value
Patient perception of residents’ empathy	1.33 (1.19, 1.47)	<0.001
Resident year of training		
PGY‐1	Reference	
PGY‐2	0.47 (0.17, 1.27)	0.138
PGY‐3	1.04 (0.40, 2.69)	0.938
Patient age	1.02 (0.99, 1.04)	0.130
Patient gender		
Male	Reference	
Female	0.77 (0.37, 1.61)	0.483
Patient races		
White	Reference	
Black	0.99 (0.40, 2.45)	0.988
Others	2.96 (0.60, 4.63)	0.184
Patient ethnicity		
Not Hispanic/Latino	Reference	
Hispanic/Latino	0.41 (0.08, 2.03)	0.278

## 4 DISCUSSION

In this study, patient perception of empathy correlates well with patient satisfaction regardless of attending physicians and residents of different training levels. In general, patient perception of attending empathy was lower than patient perception of resident empathy, especially when attending physicians working with senior EM residents. Special attention should be paid when attending physicians work with senior residents. Intrestingly, low attending empathy did not significantly affect patient satisfaction among attending physicians. In addition, we found a strong correlation between patient perception of attending empathy and patient perception of resident empathy, indicating a synergistic effect. The findings indicate that patient perception of empathy can be affected by the traditional academic provider practice model. Our study provides evidence of an academic practice pattern affecting patient perception of health care provider empathy and its correlation to patient satisfaction in an emergent care setting, which to the best of our knowledge, has not been reported before.

High JSPPPE scores correlating with high patient satisfaction has been reported in the literature and our findings are consistent with the previous reports.^19^, ^20^ Our study further determined the JSPPPE scores among EM residents of different training years (e.g., PGY1, PGY2, and PGY3) and found decreased JSPPPE scores among senior EM residents. Although no previous study reported JSPPPE focusing on EM residents, similar results can be found in a Brazil study with decreased JSPPPE scores among senior EM residents in comparison to the junior residents (interns).^18^ Other studies using different empathy tools showed a similar trend among residents of different training years.^6^, ^21^ Though we can still not fully understand the mechanism(s) for why these trends are similar, perhaps different patient illnesses (e.g., different acuity levels), patient trust in physicians or different stress levels, anxiety, or burnout among physicians could all affect physicians' empathy.^6^, ^22^ We should also consider that senior residents may have higher stress and burnout levels which could partially attribute to the decreased empathy levels^6^ as compared to their junior counterparts. Interestingly, patient perception of attending physicians empathy trended down (decreased) when the attending worked with the senior residents. Perhaps this could be partially explained as the synergistic effect occurred between the attending physicians and the residents (e.g., strong correlation between JSPPPE of attending and resident; Table 2) and that senior EM residents usually require less supervision from the attending physicians. Attending physicians trust the senior residents' performance/clinical judgement more than the junior (e.g., interns, PGY‐1) residents, for which the attending physicians may spend less time with the patients to avoid redundancies. Previous studies indicated that a provider “in‐hurry” phenomenon has an effect on patient perception of provider empathy;^17^ these findings are quite controversial^23^ and future studies are required to validate, or disprove, this phenomenon.

Patient satisfaction is a core measurement in patient‐centered care.^24^, ^25^ Though JSPPPE affects patient satisfaction, we found resident empathy and their satisfaction had less influence on patient satisfaction to attending physicians. These findings indirectly proved that patient satisfaction can be affected multifactorially, with empathy being just one contribution.^26^, ^27^ Previous studies showed many independent factors could possibly affect patient satisfaction to physicians including patient demographics, patient experience at ED, and patient trust of the physician, to list a few.^26^, ^28^, ^29^ Our study was not focused on identifying potential risks affecting patient satisfaction but rather to determine the association between JSPPPE and patient satisfaction with the influence of the attending physicians working with residents. Though, in this study, patient satisfaction was not affected significantly, caution should be taken as a down‐trending of patient perception toward the attending physician's empathy was noted. Attending physicians might need to treat patients similarly regardless of experience level of residents seeing their patients. Meanwhile, further education should be provided to senior residents on how to maintain their positive levels of empathy toward their patients. It is necessary to evaluate the current academic practice pattern affecting patient‐centered care in an emergent care setting. Our study provided limited evidence and can serve as a foundation for future studies focusing on the influence of resident academic teaching and clinical practice pattern on patient‐centered care.

### 4.1 Limitations

Our study is not without its limitations. This is a secondary data analysis derived from a single‐center prospective observational study with limited sample size. Our study setting is limited to a urban tertiary hospital with an extremely busy ED, which had patient selection bias potentially affecting its generalizability. Secondly, this study only focused on JSPPPE and patient satisfaction; we did not analyze other factors that could affect patient perception of provider empathy and patient satisfaction since these two measures can be affected multifactorially. Third, though the study showed a synergistic effect on patient perception of attending physician empathy and resident empathy and we can only address its association; more investigation is needed to determine the causative effect. Therefore, to further validate our findings, a large‐scale multicenter prospective randomized study with different patient cohorts is warranted.

## 5 CONCLUSION

Decreased patient perception of attending empathy was found when working with senior residents in comparison to attending physicians working alone or with junior residents. However, the change of patient perception of attending empathy did not significantly affect patient satisfaction towards the attending physicians.

## CONFLICT OF INTEREST

Authors have no conflict of interest.

## FUNDING

This study received no funding.

## AUTHOR CONTRIBUTIONS

Conceptualization: Ryan Kirby, Heidi C. Knowles, Nestor R. Zenarosa, and Hao Wang

Data Curation and Integrity: Ryan Kirby, Naomi Alanis, Colton Rice, and Hao Wang

Formal Analysis: Ryan Kirby and Hao Wang

Investigation: Heidi C. Knowles, Anant Patel, Naomi Alanis, and Hao Wang

Methodology: Chet D. Schrader and Hao Wang

Project Administration: Naomi Alanis and Hao Wang

Supervision: Ryan Kirby, James d'Etienne, and Hao Wang

Writing – Original Draft Preparation: Ryan Kirby and Hao Wang

Writing – Review & Editing: Ryan Kirby, Heidi C. Knowles, Anant Patel, Naomi Alanis, Colton Rice, James d'Etienne, Chet D. Schrader, Nestor R. Zenarosa, and Hao Wang.

All authors have read and approved the final version of the manuscript.

Hao Wang had full access to all of the data in this study and takes complete responsibility for the integrity of the data and the accuracy of the data analysis.

## TRANSPARENCY STATEMENT

The corresponding author (Hao Wang) affirms that this manuscript is an honest, accurate, and transparent account of the study being reported, that no important aspects of the study have been omitted, and that any discrepancies from the study as planned have been explained.

## ETHICS STATEMENT

This study was approved by the local Institutional Review Board (IRB#1352504‐6) and was performed under the Helsinki research ethics statement. All the participants signed the informed consent form.

## Data Availability

The data that support the findings of this study are available on request from the corresponding author. The data are not publicly available due to privacy or ethical restrictions.
